# Unregistered Biological Words Recognition by Q-Learning with Transfer Learning

**DOI:** 10.1155/2014/173290

**Published:** 2014-02-19

**Authors:** Fei Zhu, Quan Liu, Hui Wang, Xiaoke Zhou, Yuchen Fu

**Affiliations:** ^1^School of Computer Science and Technology, Soochow University, Suzhou 215006, China; ^2^Center for Systems Biology, Soochow University, Suzhou 215006, China

## Abstract

Unregistered biological words recognition is the process of identification of terms that is out of vocabulary. Although many approaches have been developed, the performance approaches are not satisfactory. As the identification process can be viewed as a Markov process, we put forward a Q-learning with transfer learning algorithm to detect unregistered biological words from texts. With the Q-learning, the recognizer can attain the optimal solution of identification during the interaction with the texts and contexts. During the processing, a transfer learning approach is utilized to fully take advantage of the knowledge gained in a source task to speed up learning in a different but related target task. A mapping, required by many transfer learning, which relates features from the source task to the target task, is carried on automatically under the reinforcement learning framework. We examined the performance of three approaches with GENIA corpus and JNLPBA04 data. The proposed approach improved performance in both experiments. The precision, recall rate, and *F* score results of our approach surpassed those of conventional unregistered word recognizer as well as those of Q-learning approach without transfer learning.

## 1. Introduction

From the perspective of computational linguistics, unregistered words are the ones that are out of vocabulary. They could be terms that are not documented in the vocabulary or newly generated ones. Studies on unregistered words are mainly focused on automatic recognition of them. Approaches of recognizing unregistered words are divided into rule-based approaches, statistics-based approaches, and rule-statistics hybrid approaches. Many unregistered words recognition systems have worked pretty well so far, attaining high precision in identifying general unregistered words. However, there are limited unregistered words recognition systems for dedicated domains, such as recognizer for biology terms.

Recognition of biological terms is the most important step in the extraction of biological knowledge [1], with the overall aim of identifying specific terms, such as gene, protein, disease, and drug. Numerous technologies in computing have already been employed. However, it is difficult to correctly identify biological terms in texts because they often use alphabets, digits, hyphens, and other characters [2–6]. Arbitrarily referring to biological terms makes it even harder to conduct automatic recognition. In biological text, biological named entities are usually multiword phrases and some have prefixes and/or suffixes, which makes it harder to determine the boundaries of terms. Biological terms are also affected by their context. In some cases, a biological term has a different meaning among species. As a result, it is difficult for computers to recognize biological terms automatically. Thus, general terms recognition system does not work well when they are implemented to detect biological terms.

Considering the importance and the disability of current approaches to identify unregistered words, we hereby propose a novel approach to recognize words based on transfer learning, by which we turn the process of recognizing the terms into a property marking process by redefining the property of terms according to features of terms and the corresponding context. The approach takes advantage of features of extracted candidate terms combined with transfer based error-driven learning to identify terms. By the approach, it is easier to recognize the terms with composite structure. Moreover, since the learning of the rules and feature extraction of terms rely completely on machine learning methods, it is possible to avoid the subjectivity of artificial extraction effectively and it can fit the new application well if we use a new training sample data.

## 2. Unregistered Words Recognition

The approaches of recognizing words are divided into rule-based approaches, statistics-based approaches, and rule-statistics hybrid approaches. At present, statistics-based approaches rely on frequency information of words; rule-based approaches depend on the features of the context.

The rule-based approach generates rule set or pattern base through morphological features of new coming words and identifies unregistered words by the rules or patterns. Statistics-based approach uses statistic policy to draw out candidate string and then either utilizes linguistics knowledge to exclude fake unregistered words or takes advantage of statistical analysis models, such as SVM [7–11], *t*-test [12], *n*-gram [13], HMM [14–16], CRF [17–19], neural network model [20], maximum entropy model [21–23], and other hybrid approaches [24], to find out the most relevant substring.

Generally, the rule-based unregistered words recognition system can get high recognition precision through high quality knowledge by the rules made by experts, as well as having the advantage of a small system overhead and fast running speed. However, the establishment of rules depends largely on the manual efforts, causing the difficulty of ensuring the consistency of rules. With the increasing of the rule set scale, it is getting harder and harder to carry on regular maintenance. What is more, when failing to find exactly matching rule, the system will get trouble in making an appropriate decision.

The statistics-based approaches use mathematical statistics as well as confidence of word composition to extract different kinds of knowledge for recognizing unregistered words. This kind of approaches is easy to be implemented. Combining confidence of word composition allows for considering context and experience to a larger extent. The approach turns the binary rule, true or false, to a quantification index. However, the acquisition of statistical information depends on the training corpus which needs much manual efforts. Moreover, computation cost is larger than that of rule-based approach and recognition precision is lower.

In practice, many applications use the combination of the two approaches.

We can use precision rate and recall rate to evaluate the performance of recognition evaluation. The definitions of precision rate, recall rate, and *F* scores are as follows:

(1) Precision  rate=the  number  of  correctly  identified  unregistered  wordsthe  number  of  all  identified  words,Recall  rate=the  number  of  correctly  identified  unregistered  wordsthe  number  of  all  unregistered  words,F=2∗precision  rate∗recall  rateprecision  rate+recall  rate.

## 3. Introduction to Transfer Learning

Under the conventional frame of machine learning, the objective of learning on the basis of given abundant training data is to fulfill a model for prediction. However, machine learning algorithms require great amount of training data which would cost vast manual cost and material resources.

What is more, training data and testing data are assumed to obey the identical data distribution in traditional machine learning which cannot be satisfied under many circumstances. Generally training data is likely to be overdue, which requests us to remark plenty of training data to meet our training need, which is very expensive that plentiful manual efforts and material resources have to be costive.

Therefore it is very important to fully take advantage of old training data. Transfer learning, which aims at helping learning task in the new circumstance of knowledge learned from another circumstance, can transfer knowledge from existing data to aid future learning. Transfer learning will not obey the assumption of identical distribution as traditional machine learning.

At present, the work on transfer learning can be divided into three parts: instance-based isomorphic space transfer learning, feature-based isomorphic space transfer learning, and heterogeneous space transfer learning [25], among which instance-based isomorphic space transfer learning turns out to have stronger knowledge transfer ability, feature-based isomorphic space transfer learning has broader knowledge transfer ability, and heterogeneous space transfer learning has stronger study and extension ability. Each has its own merits.

### 3.1. Instance-Based Isomorphic Space Transfer Learning

The basic idea of instance-based isomorphic space transfer learning is that there should exist partial assistant training data which is suitable to train an efficient disaggregated model and adapt to test data despite the difference between assistant training data and source training data.

Consequently, the aim of instance-based isomorphic space transfer learning is to find out instances suitable for testing data from assistant training data and transferring those instances to the study of source training data. Some researchers extended traditional AdaBoost [26] algorithm and come up with Tradaboosting [27], a boosting algorithm with transfer ability [28], to give it transfer ability to take full advantage of assistant training data to help classify the targets in the study of transfer learning based on instances.

Instance-based isomorphic space transfer learning works only when source data is extremely similar to auxiliary data. Actually, it is very difficult for instance-based isomorphic space transfer learning to find out transferable knowledge when there are great differences between source data and auxiliary data. However, source data and auxiliary data may be mixed in features level despite the fact that they cannot share some common knowledge in instances.

### 3.2. Feature-Based Isomorphic Space Transfer Learning

There are many research works on feature-based isomorphic space transfer learning, such as COCC algorithm [29], TPLSA algorithm [30], spectrum algorithm [31], and self-learning algorithm [32], which use clustering algorithm to produce a common feature for learning algorithm.

The basic thought of feature-based isomorphic space transfer learning is to utilize mutual clustering algorithm to cluster source data and auxiliary data to obtain common features which are better than features which are based on source data only and to realize transfer learning through source data of the new space. With the idea, feature-based supervised transfer learning and feature-based unsupervised transfer learning are then proposed.

The work on supervised features-based transfer learning [33] depends on mutual clustering based interdisciplinary classification. That is, how to use existed annotated data in the original filed to conduct transfer learning when there are only few sparse annotated data in the new and different field. A unified information theoretical formalized formulation is defined for interdisciplinary classification problem, among which the problems based on mutual clustering turn into optimization of destination function. In general, the objective function is defined as the loss of mutual information among source data, common features space, and auxiliary data.

The work on self-learning clustering algorithm [32] can be categorized to feature-based unsupervised transfer learning. Feature-based unsupervised transfer learning fits the case that neither of the auxiliary data of the two fields is available. Then what we have to deal with is how to utilize plenty of unannotated auxiliary data for transfer learning. The basic idea of self-learning clustering is to obtain common features through clustering on source data and auxiliary data. As new features are based on auxiliary data, the generated features tend to be better than those only from source data.

The two learning strategies introduced above solve transfer learning problem that is based on features of source data and auxiliary data in identical feature space. There is also another kind of transfer learning that is based on features across feature spaces, solving the case that source data and auxiliary data are in different spaces.

### 3.3. Heterogeneous Space Transfer Learning

Transfer learning aims at solving problems that source data and auxiliary data exist in different spaces. Lots of easily obtained annotated data are utilized to solve the problem with few annotated data.

Some work used the data with two views as a bridge to connect feature spaces of the two data spaces, which in fact acted as a translator between them. Through the translator, the nearest neighbor algorithm [34] is combined with translation features to translate auxiliary data into source data feature space. Thus generating a uniform model for learning.

### 3.4. Application of Transfer Learning in Natural Language Processing

Instance-based method in natural language processing applications was first proposed in machine translation which found out the example sentence that was the most similar with input sentence from a large-scale bilingual corpus and put the sentences in the target language and make appropriate adjustment as the input sentence translation result.

Recently, instance-based approaches for natural language processing have showed some flaws despite their pretty good behaviors. The main reason that causes them is that the longest match principle used to solve rule conflict cannot guarantee full applicability. The second reason is that a set of patterns is chosen after corpus pruning, despite having some kind of generality, and also cannot assure the correctness of results annotated according to this pattern in all cases.

Transfer-learning approach was applied to part-of-speech tagging with the same good performance as that of statistics-based approaches. The advantage of transfer-learning approach is its ability of making tagging decision on a richer event set. Moreover, some research work showed that it was easier to be understood and revised.

The advantages of transfer learning based approach can exactly make up disadvantages of instance-based approach. Therefore, our approach, based on the fundamental idea of instance-based approach, makes use of transfer learning based approach. The proposed approach obtains proper nouns through corpus and extracts relative elements which are defined as feature information of composition and structure of proper nouns from the basic proper nouns string. And then transfer learning based part draws rules from basic proper nouns string. Finally the annotation will be tagged to the candidate proper nouns.

## 4. Method

Reinforcement learning provides a framework to learn directly from the interaction and achieves goals [35, 36]. Reinforcement learning framework is abstract, flexible, and can be applied in many different applications.

In artificial intelligence field, agent is defined as an entity that has cognitive skills, the ability to solve the problem, and the ability to communicate with the outside environment. By agent, we can establish some system for controlling model. In fact, the model based on agent is an anthropomorphic model; as a result, we can control the behavior of people in the system and unify other control units, providing a unified description of the method. Agents, connected through network, act as intelligent nodes on the network, therefore constructing a distributed multiagent system.

**Figure 1:**
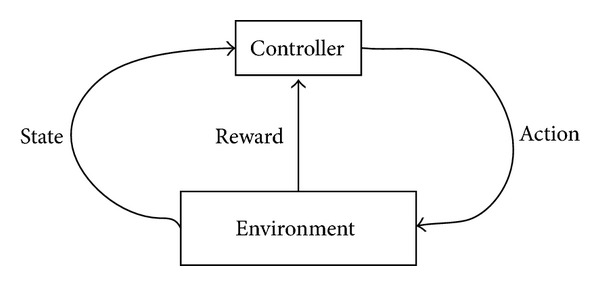
Framework of reinforcement learning. Controller selects an action; the environment responds to the action, generates new scenes to the agent, and then returns a reward.

In reinforcement learning framework, an agent, named as controller, is a learner and decision-maker, interacting with environment which is outside of agent. Controller chooses an action; the environment responds to the action, generates new scenes to the agent, and then returns a reward. The framework [35, 37, 38] of reinforcement learning is showed as Figure 1.

Controller interacts with the environment at each step during a discrete-time sequence (*k* = 0,1, …). At each time step *k*, agent gets the representation of environment denoted by state *x*_*k*_ ∈ *X*, where *X* is the set of all possible states; controller chooses an action *u*_*k*_ ∈ *U* according to its policy *h* : *X* → *U* using *u*_*k*_ = *h*(*x*_*k*_), where *u* is all available actions. By taking the action, agent receives a reward $\begin{array}{cc} r_{k+1}=\rho \left(x_{k},u_{k}\right) & \rho :X\times U\to R \end{array}$ and gets to a new status *x*_*k*+1_ [39]. The ultimate goal of controller is to maximize the sum of the rewards in long term. The mapping from state to action selection is policy of the agent policy, denoted by *π*_*t*_. Reinforcement learning solves how agent changes policy through experience.

The temporal difference (TD) learning is capable of learning directly from raw experience without determining dynamic model of environment in advance [36, 38]. Moreover, the model learned by temporal difference is updated by estimation which is based on part of learning rather than final results of the learning. These two characteristics of temporal difference make it particularly suitable for solving the prediction problems and control problems in real-time control applications. Given some experience with policy *h*, temporal difference learning updates estimated *V* of *V*_*h*_ [40], as

(2) $$\begin{array}{c} V\left(x_{k}\right)⟵V\left(x_{k}\right)+\alpha \left[R_{k}-V\left(x_{t}\right)\right], \end{array}$$

where *R*_*k*_ is the actual return after time step *k* and *α* is a step size parameter. Temporal difference learning updates *V* in step *k* + 1 using the observed reward *r*_*k*+1_ and estimated *V*(*x*_*k*+1_).

Let *Q* ^*h*^(*x*, *u*) be the value of taking action *u*, in *U* under a policy. *Q* ^*h*^(*x*, *u*) [41] is defined as

(3) $$\begin{array}{c} Q^{h}\left(x,u\right)=\rho \left(x,u\right)+\overset{\infty }{\underset{k=1}{\sum }}\gamma^{k}\rho \left(x_{k},u_{k}\right). \end{array}$$

Q-learning is an off-policy version of TD control, which is defined by

(4) Q(xk,uk)⟵Q(xk,uk)+α[rk+1+γmax⁡u Q(xk+1,u)−Q(xk,uk)].

The term identification process is actually the determination process of which kind of label should be tagged to a word, and thus can be viewed as Markov processes, denoted by 〈*X*, *U*, *R*〉, where *X* represents the state of tagging, *U* stands for the action by the controller, and *R* indicates the return attained.

### 4.1. Definition of State

**Definition 1**

Proper noun feature word (F) is the word that reflects the categorization character of the unregistered word. There are prefix feature word (PF), intermediate feature word (IF), and suffix feature word (SF) according to the different position of the feature word in the proper noun.

**Definition 2**

Conjunctive word (J) is the conjunction part of a proper noun word to connect the words.

**Definition 3**

Word boundary (B) denotes the boundary word of proper noun word and its contexts. The left word boundary (LB) represents the previous context of the word and the right word boundary (RB) is the following context of the word.

**Definition 4**

Other word (O) is the word that is not any part of proper noun.

**Definition 5**

Fundamental proper noun string (FPNS) is the string that consists of the elements as defined in Definition 1 to Definition 4.

Hereby we define seven states for a candidate term, as listed in Table 1.

### 4.2. Definition of Action

In reinforcement learning framework, policy defines the learning agent behavior at a given time. It in fact is a mapping from perceived states to available actions. Reinforcement learning model obtains rewards by mapping the scene to the action which affects not only the direct rewards, but also the next scene, so that all subsequent rewards will be influenced. Specific states and actions are very different in various applications.

**Definition 6**

Positive rules are those by which features are determined as proper nouns.

**Definition 7**

Negative rules are those by which features are not determined as proper nouns.

**Definition 8**

Neuter rules are those by which features are not determined as proper nouns.

We refer the feature with information valid value less than the average valid value as low information value and the one greater than the average as high information value.

We extract positive rules from features with low information value supplemented by extracting negative rules and draw negative rules from features with high information value supplemented by drawing positive rules. The advantage of using this policy is that we can control the total number of rules and thus spare searching space and storage space.

We define three actions that a controller can choose in a certain state, as in Table 2.

### 4.3. Definitions of Reward and Return

Reward function in reinforcement learning defines the goal of the problem. The perceived state of the environment is mapped to a value, reward, representing internal needs of the state. The ultimate goal of reinforcement learning agent is to maximize the total reward in long term.

In our work, controller makes decisions under different combinations of the word-annotation pair, so that by the actions we can maximal correct tagged unregistered words. Here, we use annotation quality indicator to evaluate the behavior. Given a feature, we use valid annotation value to score the quality of the feature as

(5) $$\begin{array}{c} \text{Quality}\left(f,t\right)=F\;\text{score}\;\text{a}\;\text{feature}\;f\;\text{with}\;\text{tag}\;t. \end{array}$$

The reward of tagging is given by:

(6) $$\begin{array}{c} r=\text{Quality}\left(f,t\right),\\ Q^{h}\left(x,u\right)=r\left(x,u\right)+\overset{\infty }{\underset{k=1}{\sum }}\gamma^{k}r\left(x_{k},u_{k}\right). \end{array}$$

### 4.4. Transfers Learning

**Figure 2:**
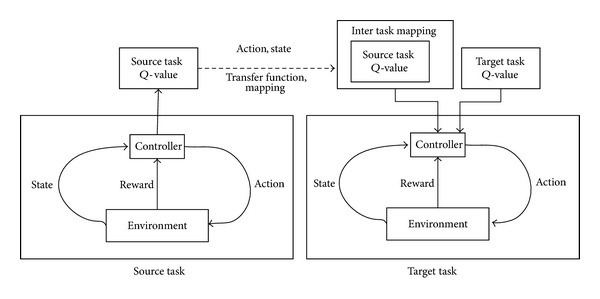
Framework of reinforcement learning with transfer learning. The *Q*-value from the source tasks is also involved in the target tasks through transfer function.

Transfer learning involves reusing knowledge learned from earlier tasks to learn new problems more effectively. The task learned previously is called the source task and the new task is called the target task.  Figure 2 shows how the action-value *Q*-value reuses the empirical works of the source corpus.

We use *Q*-value reuse for the transfer, where the action-value function, *Q*-source, learned from the corpus is used as a starting point for the new problem, and a new action-value function, *Q*-target, is learned to correct errors in the source action-value function. However, the source state and action spaces may not coincide with the target state and action spaces. Therefore, the controller must be given a mapping between the source and target tasks. Therefore, the controller's new *Q* function is given by

(7) $$\begin{array}{c} Q\left(x,u\right)=Q_{\text{source}}\left(f_{x}\left(x\right),f_{u}\left(u\right)\right)+Q_{\text{target}}\left(x,u\right). \end{array}$$

The goal of transfer learning algorithms is to utilize knowledge gained in a source task to speed up learning.  Algorithm 1 generates a transfer function for reinforcement learning.

### 4.5. Unregistered Words Identification by Q-Learning with Transfer Learning

The processing flow unregistered words by Q-learning with transfer learning is showed as follows.

**Step 1 (Tagging initially)**

Use an initial tagger machine to annotate the training corpus.

**Step 2 (Generating a set of candidate rules)**

For each incorrect term, the rule template will be used to generate candidate rules. The state of the rule condition is the context of the word and the action is to amend the incorrect tags.

**Step 3 (Attaining rules)**

Apply each rule in candidate rule set to annotated corpus so as to get a tagging result, and compare the result with the standard answer, and then get the rule with the high evaluation score. Use the result returned by the rule as the basis of next iteration, and assign the rule with the highest priority.

**Step 4**

Repeat the above steps until the evaluation score is less than a predefine threshold.

An ordered rule set will be generated through the above automatic learning process. By the approach, we can use more syntactic and semantic rule in a wider range. Particularly, the tagging can be built on the basis of word and its corresponding context. The transfer-based tagging requires much less computation than most of Markov-based model. What attracts us the most is that the transfer-based approach is free from over training which is suffered by most hidden Markov models.

## 5. Experiment and Results

### 5.1. Materials

There are numerous benchmark corpuses for biological terms identification, such as the GENIA [42] data set and JNLPBA04 shared task data set [43]. The GENIA corpus contains 2,000 MEDLINE abstracts with more than 400,000 words and almost 100,000 annotations of biological terms [42]. JNLPBA04 [43] has several shared tasks for natural language processing in biomedicine and its application. Both data sets are often used as benchmark data sets for evaluation.

**Figure 3:**
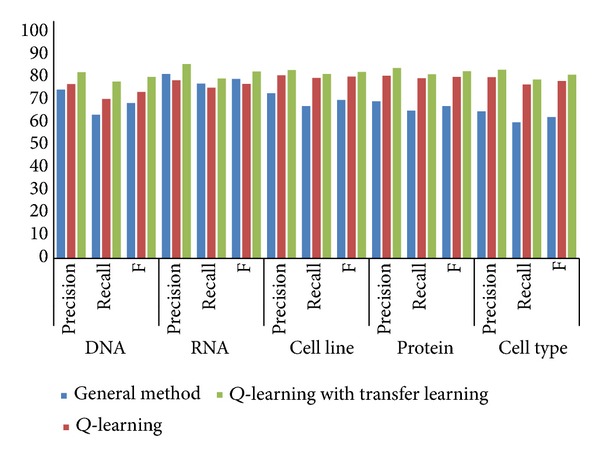
Precision, recall rate, and *F* score results of identifying four kinds of unregistered words: DNA, RNA, cell line, protein, and cell type from GENIA.

We carry on two rounds of testing. In the first round of testing, we randomly selected terms from GENIA corpus and divided them into two parts, one part for training and the other for testing. In the experiment, we identify four kinds of unregistered biological terms: DNA, RNA, cell line, protein, and cell type. And we use precision, recall rate, and *F* score to evaluate the results. The results are showed as Figure 3.

**Figure 4:**
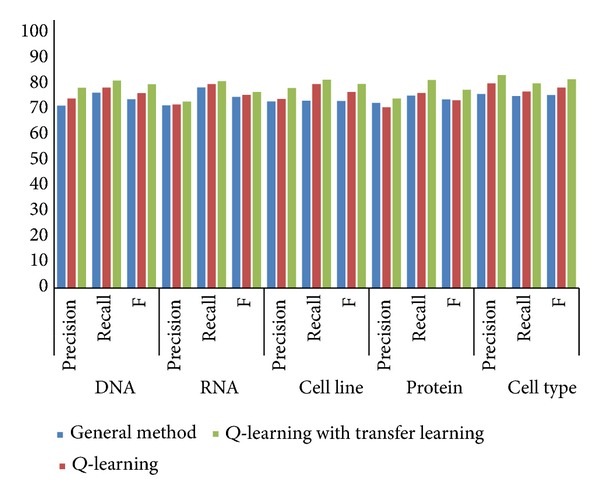
Precision, recall rate, and *F* score results of identifying four kinds of unregistered words: DNA, RNA, cell line, protein, and cell type from JNLPBA04.

In the second round of testing, we randomly selected terms from JNLPBA04 and divided them into two parts, one part for training and the other for testing. The results are showed as Figure 4.

We can see from both results that the Q-learning based recognizer is generally better than the general unregistered word recognizer, but not for all cases. This is because that Q-learning tries the policy that can get the best identification effort to the controller's knowledge. However, it does not always work as the controller may fall to a local optimal solution. When we add knowledge by transfer learning, the recognizer gains a remarkable improvement in the three evaluation factors. Hereby we can say that Q-learning with knowledge transfer learning approach is the best of the three.

## 6. Conclusion

Huge numbers of biological texts provide us with a highly reliable information source for biological research. How to mine information and find new knowledge efficiently and effectively are a very important new issue to researchers. Recognizing unregistered biological words from texts is essential to biological text mining.

In this work, we propose unregistered biological words. This approach used Q-learning algorithm so as to attain optimal solution to choose the tag for the terms and took advantage of transfer learning to fully take advantage of existing knowledge (Algorithm 2).

We carried on two rounds of testing on three approaches. In the first round testing, three recognizers identified unregistered words from GENIA corpus, and in the second round of testing, they identified unregistered words from JNLPBA04 corpus. Both of the testing results showed that the approach by Q-learning algorithm with transfer learning, was the best of the three. It really improved the performance of unregistered biological words identification.

## Figures and Tables

**Algorithm 1:**
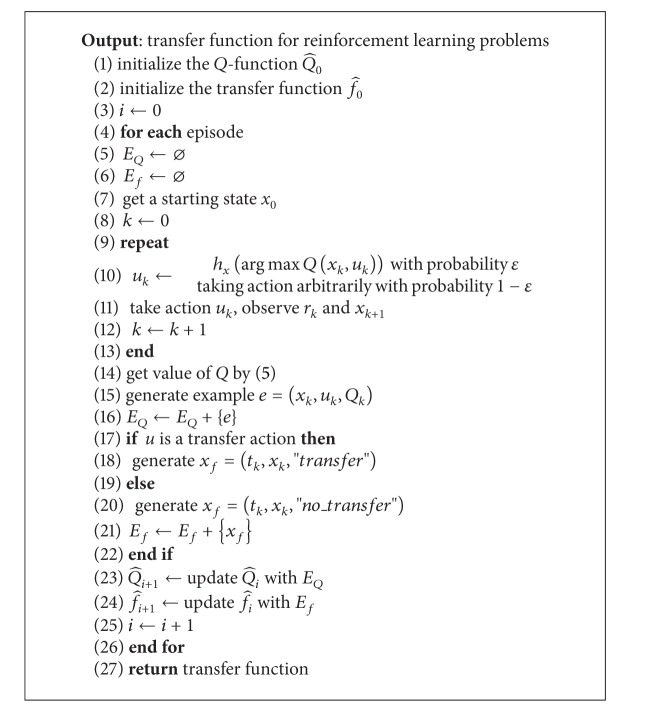
Transfer function learning.

**Algorithm 2:**
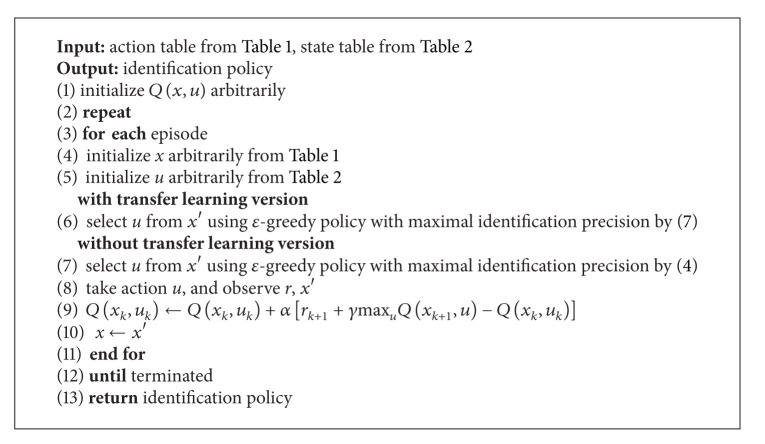
Unregistered biological terms identification by Q-learning with transfer learning.

**Table 1:** States for a candidate term.

Value	State	Comment
0	PF	Prefix feature word
1	IF	Intermediate feature word
2	SF	Suffix feature word
3	J	Conjunctive word
4	LB	Left word boundary
5	RB	Right word boundary
6	O	Other words

**Table 2:** Actions for a candidate term.

Value	Action
0	Positive rule decision
1	Negative rule decision
2	Neuter rule decision
